# Disseminated Histoplasmosis in an Indigenous Child With Malnutrition: A Case Report

**DOI:** 10.7759/cureus.41493

**Published:** 2023-07-07

**Authors:** Ioanna I Yglesias Dimadi, Madelyn Clinton Hidalgo, Vivian I Hernández Chavarría, Hery Min Kim, Grettel R Castro Torres

**Affiliations:** 1 General Medicine, Universidad de Costa Rica, San Jose, CRI; 2 Pediatric Medicine, Caja Costarricense del Seguro Social, San Jose, CRI

**Keywords:** immunodeficiency, disseminated disease, hepatosplenomegaly, fungal infection, malnutrition, histoplasmosis

## Abstract

Histoplasmosis is a mycosis caused by *Histoplasma capsulatum*, a dimorphic fungus endemic to areas with nitrogen-rich soil, like the one contaminated with bird and bat excrement. Patients with a deficient immune response are especially at risk for developing invasive infections, such as disseminated histoplasmosis, and secondary immunodeficiency can be a consequence of malnutrition. This case report presents a 15-month-old male infant with malnutrition who presented with signs and symptoms of disseminated histoplasmosis, including fever, malaise, weight loss, cough, and diarrhea. The infant came from a geographic area where histoplasmosis is endemic, and he was a member of a cultural group with a higher prevalence of histoplasmosis than the general population. On physical examination, hepatosplenomegaly, lymphadenopathy, and lung crackles were found, which are common in most patients with histoplasmosis. The keystone of diagnosis of *H. capsulatum* infection is antigen detection, but the criterion standard is isolation of the organism from body specimens through laboratory culture. Histological diagnosis is especially useful for rapid diagnosis. Treatment of disseminated histoplasmosis in the pediatric population consists of deoxycholate amphotericin B for four to six weeks followed by itraconazole to complete a total of three months of treatment. Despite the involvement of multiple organ systems, the patient recovered satisfactorily after the completion of amphotericin B treatment for one month and the resolution of his malnourishment.

## Introduction

Histoplasmosis is a common fungal infection caused by the soil-based Ascomycete fungus *Histoplasma capsulatum*, a dimorphic fungus that exists in two forms: the mycelial form, which is found in nature at ambient temperatures, and the yeast, or infectious, disease-causing form, which exists at body temperature [1].* H. capsulatum* is naturally found in nitrogen-rich soil, such as soil contaminated by guano, a mixture of soil and decaying bird excrement. Highly infectious soil in endemic areas is characterized by an acidic pH, temperature between -18 and 37°C, 12% moisture, and high concentrations of carbohydrates and cationic salts. These characteristics are typical in areas inhabited by bats and birds [2].

Excavation and construction disrupt soil that contains guano and are associated with the dispersal of the fungus into the environment. Airborne transmission between humans has never been reported. Sex distribution shows it is four times more common in males than in females [1]. Most cases are diagnosed in HIV patients, and it is considered an AIDS-defining opportunistic illness [3].

Histoplasmosis infection has been repeatedly reported in Central and South American countries, including Panama, Guatemala, Nicaragua, El Salvador, and Costa Rica. According to a study performed by Gascón et al. [4], as cited by Scully and Baddley [3], around 20% of travelers returning to Europe from Central and South America had a positive *Histoplasma* skin test one month after their return [3,4]. In the specific case of Panama, Gomez BL [5] reported that prevalence can reach as high as 50% in endemic areas based on the results of histoplasmin reactivity tests. In Costa Rica, acute pulmonary disease and disseminated histoplasmosis have been reported [5].

An important sociocultural group particularly affected by histoplasmosis is the Ngäbe-Buglé indigenous group, a population originally from Panama that has migrated to Costa Rica in the past several years to work in coffee plantations. Among their social problems are extreme poverty, high maternal and infant mortality rates, inhumane working conditions, drug use, and migratory irregularities [6]. This group is especially affected by histoplasmosis because of their settlement in histoplasma-rich territories and because of their socioeconomic activities which usually include plantation, collection, and contact with poultry. 

Studies and case reports of disseminated histoplasmosis in children in Costa Rica are scarce. In a retrospective observational study, Batalla S [7], reviewed 18 cases of pediatric disseminated histoplasmosis in the National Children’s Hospital in Costa Rica. Seventy-two percent of the patients were male, with a mean age of 12 months; 44% were underweight; and 78% had a positive epidemiological nexus with chicken and bats. The most common clinical manifestations at diagnosis were hepatomegaly (89%), splenomegaly (83%), and fever (78%); however, only 39% of the patients presented with respiratory symptoms.

Histoplasmosis infection can present in one of two forms: acute primary infection or progressive disseminated histoplasmosis (PDH). In the acute form, most individuals have an asymptomatic course, but it can also manifest with high fever (up to 42°C), headache, nonproductive cough, and chest pain due to enlargement of mediastinal lymph nodes. Risk factors for developing symptoms are a large inoculum, age (older adults and children under 2 years), and being immunocompromised. Rheumatologic symptoms occur in a minority of patients, mostly women. Some signs, if present, include crackles on auscultation, hepatosplenomegaly, and patchy infiltrates on chest X-ray. Laboratory tests might show leukocytosis or leucopenia and increased alkaline phosphatase [1,8].

PDH is very rare. It is characterized by the growth of *H. capsulatum* in multiple organ systems, and most cases are seen in immunosuppressed individuals (those with HIV/AIDS, solid organ recipients, bone marrow transplantation, and patients in TNF-alpha therapy). However, it can occur in previously healthy individuals at the extremes of age (>55 days and <1 year) with no known underlying immunologic dysfunction [9,10]. This form can develop from either re-exposure to the fungus or reactivation of a latent previous infection. Usually, progressive histoplasmosis develops because of an impaired cell-mediated immune response against the organism [11].

Currently, the mainstay of treatment for histoplasmosis is antifungal therapy. In the case of acute disseminated histoplasmosis in children, the treatment consists of deoxycholate amphotericin B (1 mg/kg daily) for four to six weeks followed by itraconazole (5-10 mg/kg daily) for a total of three months of treatment [1]. In the past, before antifungal treatments were established, mortality among untreated children was as high as 100% in five weeks, usually due to disseminated intravascular coagulation, gastrointestinal bleeding, and secondary bacterial infection [1].

An important information gap exists regarding histoplasmosis in the pediatric population. Most of the available information consists of case reports and case series. Although the link between disseminated histoplasmosis and immunosuppression is widely accepted, no literature was found that specifically associated this form of the disease with malnourishment. The current case involved a near-fatal clinical presentation of disseminated histoplasmosis in an infant with malnourishment and no other underlying clinical conditions. This article is intended to provide up-to-date information for making a clinical diagnosis in similar situations and to encourage starting treatment promptly to avoid mortality or morbidity. This article portrays the real struggle that represents making the prompt diagnosis of disseminated histoplasmosis in children with no other known medical condition, especially in resource-limited healthcare centers.

## Case presentation

We present the case of an infant with severe chronic malnutrition and severely underweight and disseminated histoplasmosis. The patient was a 15-month-old boy born in Panama and a member of the Ngäbe (JRB1)-Buglé indigenous group. He was residing in Costa Rica when he became ill. He had no medical history of importance. His immunization schedule was complete for his age according to the Panamanian and Costa Rican schedules.

The child first presented to the emergency department of a peripheral hospital of the Caja Costarricense del Seguro Social (JRB2) of Costa Rica, with a three-month history of cough, intermittent fever, and constipation. The physical examination revealed that all anthropometric measurements (weight for age, height for age, and head circumference for age) were below the third percentile. The weight-for-height measurement was within the normal range. The patient presented clear signs of chronic malnutrition, with decreased fatty tissue in his extremities and trunk, dry skin, thin hair, and diffuse hair loss patches. In addition, he also presented with notable hepatosplenomegaly extending to the iliac fossae and bilateral basal rhonchi. Upon admission, the patient required supplemental oxygen because of persistent hypoxia.

Laboratory testing showed anemia, thrombocytopenia, lymphopenia, electrolytic disturbance, hypoalbuminemia, and altered liver function tests. Renal function was normal. Table 1 shows the patient’s laboratory values compared with normal laboratory values of the pediatric population.

**Table 1:** Laboratory values of the patient upon admission

Laboratory test	Patient’s value	Normal values for an infant 1–2 years old [12,13]
Hemogram values		
Hemoglobin	8.1 g/dL	12 g/dL
Platelets	64,000/µL	200,000-400,000/µL
Lymphocytes	1200/µL	4000-10,500-/µL
Metabolic values		
Albumin	2.8 g/dL	Hypoalbuminemia <3.5 g/dL
Alkaline phosphatase	201 IU/L	Values vary by laboratory
Aspartate aminotransferase	41 U/L	

**Figure 1:**
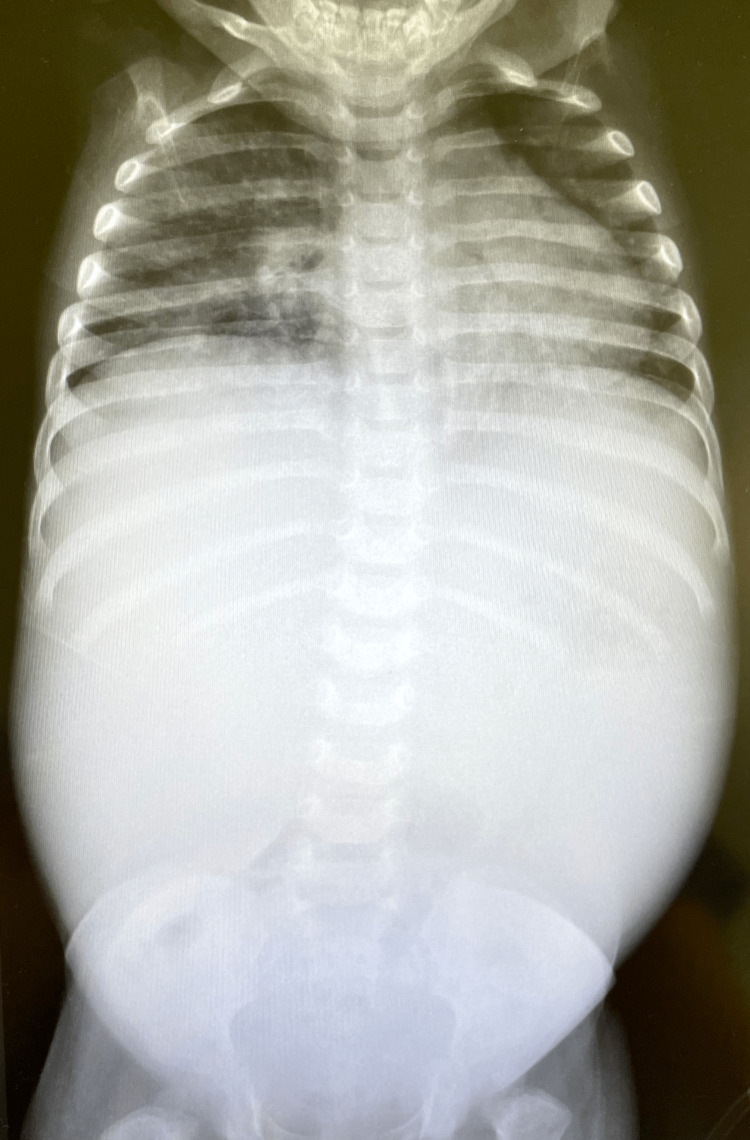
Abdominal X-ray with hepatosplenomegaly Note the radiopaque silhouette of the liver and spleen extending from the diaphragm to the iliac fossae. Note also how the thoracic cavity is reduced in size secondary to the enlarged viscera.

An abdominal ultrasound performed to study the hepatosplenomegaly showed diffuse enlargement of the liver and spleen without focal and cystic lesions. The longitudinal diameter was 11 cm for the liver and 10 cm for the spleen. The sonographic image was not compatible with fatty liver (unfortunately, only the ultrasound report was available, not the images). The abdominal X-ray in Figure 1 shows radiopaque hepatosplenomegaly without focal or cystic lesions.

**Figure 2:**
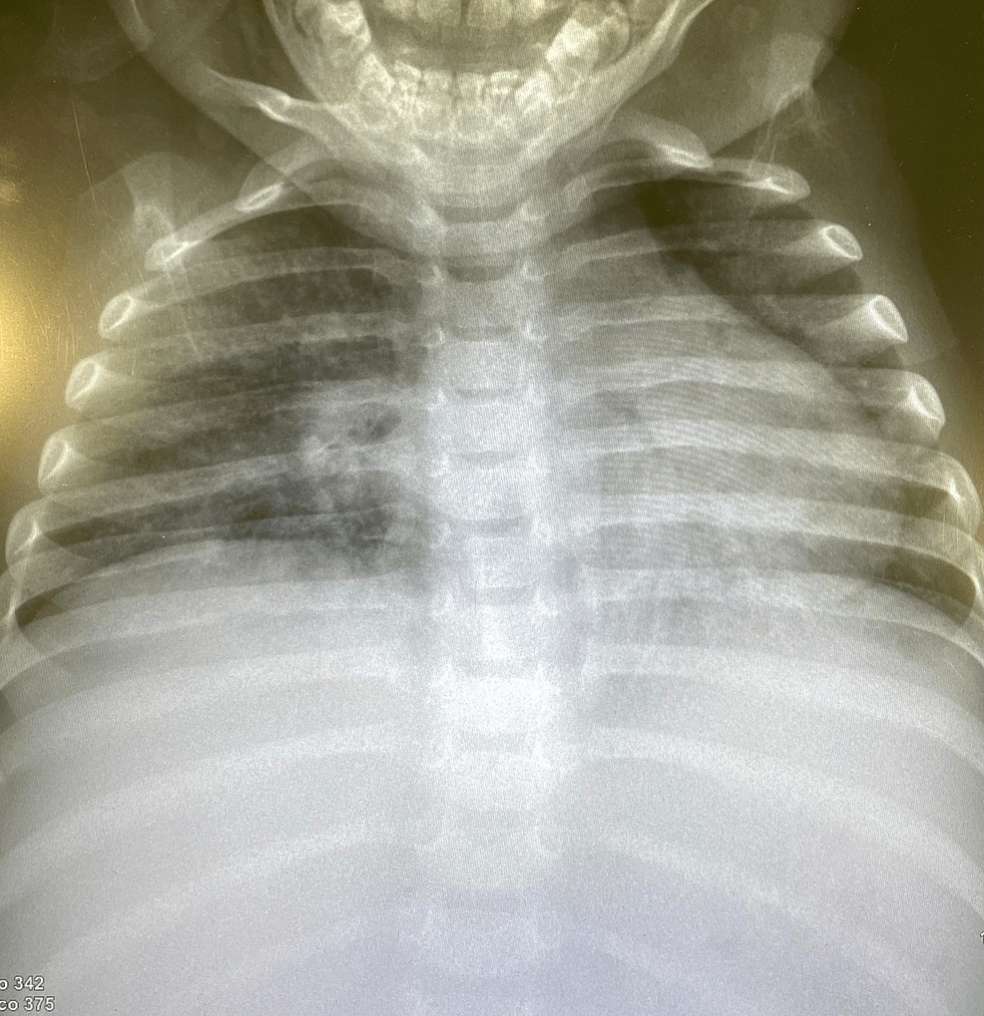
Chest X-ray image Note the increased parahilar vascularity with a parahilar infiltrate in the right lung.

Owing to respiratory symptoms, the patient underwent a complete respiratory study. Chest X-ray (Figure 2) showed increased parahilar vascularity summed to a parahilar infiltrate in the right lung. Immunofluorescence was positive for metapneumovirus and type 4 *parainfluenza virus*. A bronchoscopy was performed, which proved purulent tracheitis. The bronchoalveolar lavage was positive for methicillin-sensitive *Staphylococcus aureus* (MSSA). Based on the patient’s sociocultural background, the bronchoalveolar lavage was cultured for mycobacteria and fungi. Antibiotic therapy with clindamycin was indicated based on the antibiogram of the MSSA. 

The results of the physical examination, along with clinical history and laboratory findings, led to bone marrow aspiration to rule out leukemia. It was negative for leukemic infiltration, but it showed decreased thrombopoiesis, suggestive of infiltrative infection by histoplasmosis.

Other tests, including lumbar puncture for central nervous system analysis and multiple viral serologies, were performed to rule out differential diagnosis possibilities. Polymerase chain reaction (PCR) analysis of the cerebrospinal fluid was negative for Epstein-Barr virus, cytomegalovirus, herpes 6, herpes 7, herpes simplex virus 1, herpes simplex virus 2, parvovirus, paramyxovirus, varicella zoster virus, and parechovirus. Only serum Epstein-Barr immunoglobulin (Ig)M had a positive result. The enzyme-linked immunoassay (ELISA) for HIV (JRB1) was negative, and no other HIV tests were performed at the moment due to the low grade of suspicion at the time. 

Despite the antibiotic coverage, the patient continued to have respiratory difficulty, hypoxia, fever, and cough after several days of treatment. Ziehl-Neelsen staining and polymerase chain reaction (PCR) analysis for *Mycobacterium tuberculosis* of the bronchoalveolar lavage (BAL) were negative, and cultures of the BAL for this pathogen remained negative after four weeks, decreasing the possibility of tuberculosis. Although PCR for *H. capsulatum* in the same BAL was negative, the cytological smear showed budding oval yeasts compatible with histoplasmosis.

Because of the involvement of the respiratory and hematopoietic systems, along with infiltration of the spleen, liver, and bone marrow, the diagnosis of systemic histoplasmosis was made. Based on this diagnosis, amphotericin B deoxycholate 1 mg/kg/day intravenous (IV) was started and continued for 30 days. After one week of this treatment, methylprednisolone was given for seven days to reduce systemic inflammation. Within one month of antifungal therapy, the patient’s respiratory symptoms and pancytopenia were resolved. The hepatosplenomegaly disappeared almost completely. The treatment was then changed to oral itraconazole to be continued for 11 months to complete a total of one year of antifungal therapy. The patient was then discharged to continue oral treatment at home with careful monitoring of liver function.

**Figure 3:**
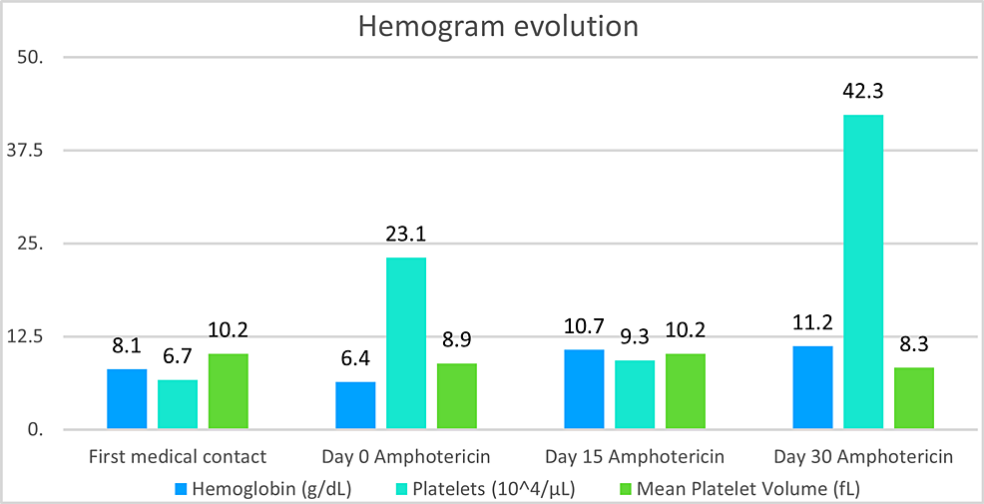
Changes in hemogram values through time Note how the patient presented with anemia and thrombocytopenia at his first medical contact. At day 0 of amphotericin, the patient had a hemoglobin value of 6.4 g/dL, which required a transfusion of one unit of packed red blood cells. After completing one month of amphotericin, the patient’s anemia and thrombocytopenia were resolved, which was probably associated with bone marrow reactivation after histoplasmosis eradication.

**Figure 4:**
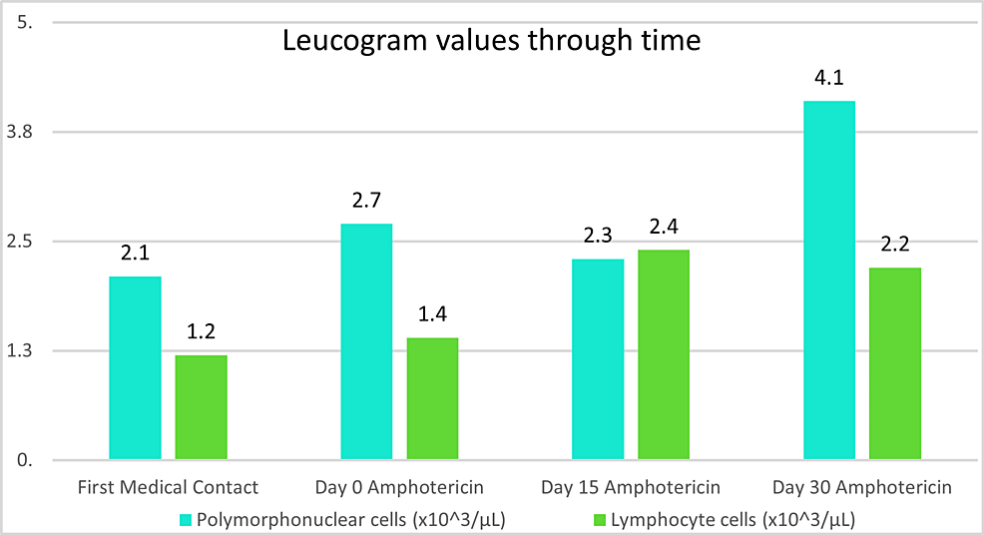
Changes in leucogram values through time Note how leucocyte values rose as the patient completed amphotericin treatment and as malnourishment was resolved.

**Figure 5:**
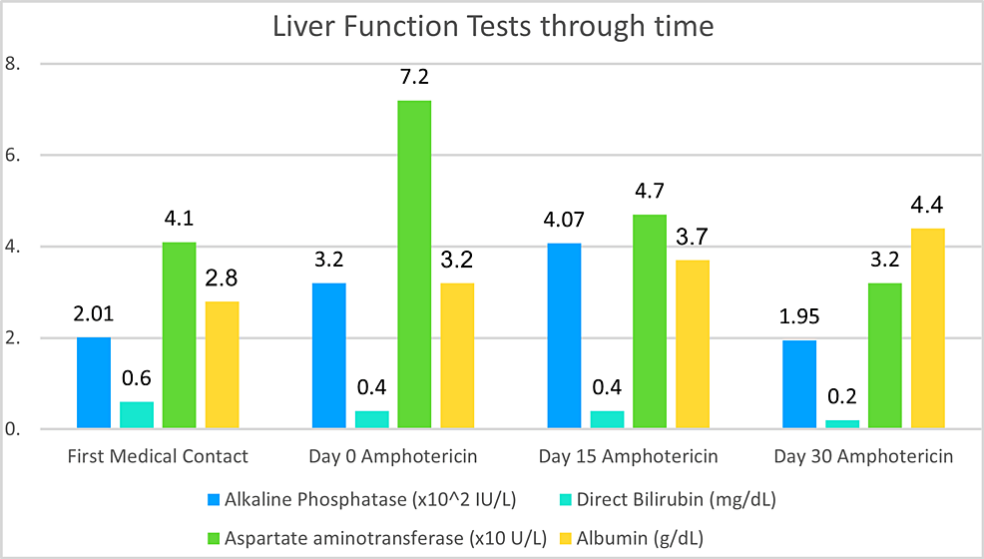
Liver function tests and albumin levels through time. Note how liver function tests normalized as histoplasmosis resolved. Note how albumin levels rose during the treatment course as the patient was placed on a high-protein hypercaloric diet.

The following Figures 3-5 present the evolution of the patient’s laboratory tests from his first medical contact, through his medical treatment, and at the end of the 30-day amphotericin regimen.

## Discussion

In the case presented above, the patient’s sociocultural background is of special importance. This population is commonly exposed to risk factors because they usually live in houses with dirt floors surrounded by poultry, and histoplasma-contaminated guano is common. Also, due to economic constraints, the patient’s nutrition was deficient for his growth state, causing him to be severely malnourished.

According to the American Society of Parenteral and Enteral Nutrition as cited in Dipasquale et al. [14] study, the imbalance between the requirement and intake of energy arising from pediatric malnutrition can lead to growth restriction, muscle and fat loss, and a decreased metabolic rate. These effects were objectively recorded in our patient’s anthropometric measurements, which were below the third percentile. In addition, factors that have been associated with a higher risk for malnutrition are age under 1 year, low socioeconomic status, and ethnic/racial minority [15], as in our patient.

One of the consequences of malnutrition is the development of secondary immunodeficiency, increasing the risk of opportunistic infections, the need for parenteral antibiotics, and disease caused by uncommon etiologic pathogens. Further, infections perpetuate malnutrition because of decreased food intake and increased energy use. One characteristic feature that distinguishes malnutrition-related immunodeficiency from other secondary immunodeficiencies is the restoration of a normal immune response with adequate nutrition [16].

Pathogenesis of *H. capsulatum*

In human infection, *H. capsulatum* is liberated in mycelial form from soil and enters the body through the respiratory tract. Conidia are then phagocytized by neutrophils and macrophages. The conversion from mycelial to yeast form, which is crucial for the infection to be established, occurs in the intracellular environment [1,2].

Although cellular immunity may contain the infection, yeast can remain dormant in the infected tissues. This latent infection causes no harm in an immunocompetent host and may be present without symptoms for years. However, immunosuppression leads to the activation of the dormant infection, causing the progressive disseminated form of the disease [1]. Our patient may have had dormant histoplasmosis that became active secondary to his malnourishment and subsequent immunocompromised status.

Clinical presentation

One specific form of PDH, the acute PDH form, was previously called the infantile form because of its predominance in this age group. Currently, it is also seen in severely immunosuppressed patients, such as those with AIDS and hematologic malignancies. Symptoms include fever, malaise, weight loss, cough, and diarrhea. On physical examination, most patients are found to have hepatosplenomegaly, lymphadenopathy, and pulmonary crackles [1,17].

Although our patient presented with some symptoms of acute primary infection, his multiorgan compromise (liver, spleen, lungs, and bone marrow) was characteristic of the progressive disseminated form. It is likely that the patient’s age and malnourishment predisposed him to this form of the disease, but we cannot rule out congenital immunodeficiency because the necessary tests could not be performed during the acute phase of the disease. Further, the patient was lost to follow-up after hospital discharge. 

Diagnosis

The diagnosis of *H. capsulatum* typically relies on antigen detection in urine or serum specimens; but can also be done in BAL fluid or cerebrospinal fluid. Antigen tests are also useful to monitor response to treatment. The criterion standard for diagnosis is the isolation of the organism from body specimens through culture. The use of PCR analysis for the detection of *H. capsulatum* is still under study. Serology testing has also been described, but its use is more for retrospective diagnosis and in the subacute or chronic forms. Further, serology results can be negative in up to 50% of patients with immunosuppression, limiting its use. Histological diagnosis of *H. capsulatum* is especially useful for rapid diagnosis. Cytologic diagnosis is useful in disseminated histoplasmosis when using samples such as peripheral blood smear, bone marrow smear, and BAL fluid and samples collected by fine-needle aspiration [1,18].

Complementary tests

In acute progressive disseminated histoplasmosis, laboratory tests are usually altered. Around 90% of patients have anemia, and 80% of affected children present with leucopenia and thrombocytopenia. Patchy infiltrates with mediastinal and hilar lymph node enlargement are frequently found on chest X-rays [1,17]. When the liver is affected, although transaminases may be elevated, high levels of alkaline phosphatase (≥2100 U/L) are one of the most prominent biochemical laboratory findings [19]. 

Differential diagnosis 

In our case, the initial differential diagnosis included lymphoproliferative disease, based on the findings of fever, pancytopenia, and diffuse hepatosplenomegaly [20]. However, bone marrow testing ruled out this possibility. The diagnosis was difficult in our setting. We did not conduct *H. capsulatum* antigen testing, and the initial PCR test performed in BAL was negative. Further, BAL was cultured for *H. capsulatum*, but the patient’s declining condition precluded waiting two to four weeks for the results. The final diagnosis was made based on clinical presentation, patient history, and cytology of BAL suggestive of histoplasmosis. This diagnosis was further confirmed by the patient’s clinical improvement after initiating amphotericin 1 mg/kg/d IV, which coincides with a fungal underlying etiology. After one month, the patient was changed to itraconazole, to be continued for 11 months and not two months as normally recommended because of our concerns for the patient’s immunologic status, sociocultural environment, and adherence to treatment.

## Conclusions

*H. capsulatum* is a fungus normally found in soil contaminated with guano in areas inhabited by bats and birds. Most cases of progressive disseminated histoplasmosis occur in immunosuppressed individuals or those at extremes of age. This form of the disease is highly fatal without prompt recognition and initiation of treatment. Disseminated disease can be suspected with the presence of hematologic alterations, hepatosplenomegaly, and pulmonary affection. The keystone of diagnosis is antigen detection in body specimens. Treatment must be started promptly with amphotericin B and then titrated to itraconazole to complete in three months.

Among the factors that possibly negatively affected the development of the case were not performing a PCR test for HIV after the ELISA test was negative, not conducting an antigen test, and relying on nonstandard diagnostic techniques. Limitations faced during this research include the limited bibliography concerning histoplasmosis and the pediatric population and the inability to obtain better-explaining pictures of the patient’s diagnostic imaging tests. More information and studies are needed to understand better the clinical presentation, risk factors, and prognosis of disseminated histoplasmosis in the pediatric population. Finally, recording the patients’ nutritional status in the setting of disseminated histoplasmosis could help to establish a relationship between this pathology with malnutrition, especially in the pediatric population.
